# Papaverine Mitigates Acute Kidney Injury in Feces-Induced Polymicrobial Sepsis Through Regulation of the HMGB1–RAGE Axis

**DOI:** 10.3390/medicina62040621

**Published:** 2026-03-25

**Authors:** Mehmet Fatih Dasiran, Ahmet Akbaş, Bakiye Akbaş, Ejder Saylav Bora, Hatice Aygun, Oytun Erbas

**Affiliations:** 1 Department of General Surgery, Faculty of Medicine, Tokat Gaziosmanpasa University, Tokat 60250, Türkiye; fatihdasiran@yahoo.com; 2 Department of General Surgery, Faculty of Medicine, Karadeniz Technical University, Trabzon 61080, Türkiye; draakbas@hotmail.com; 3 Department of Obstetrics and Gynecology, Faculty of Medicine, Karadeniz Technical University, Trabzon 61080, Türkiye; bakiyeakbas@ktu.edu.tr; 4 Department of Emergency Medicine, Faculty of Medicine, Izmir Katip Çelebi University, İzmir 35100, Türkiye; saylavbora@hotmail.com; 5 Neuroscience Laboratory, BAMER, Biruni University, Istanbul 34015, Türkiye; 6 BAMER, Biruni University, Istanbul 34015, Türkiye; oytunerbas2012@gmail.com

**Keywords:** papaverine, kidney, sepsis, HMGB1–RAGE, feces-induced peritonitis

## Abstract

*Background and Objectives*: Sepsis-associated acute kidney injury (SA-AKI) is driven by exaggerated inflammation and oxidative stress, with the HMGB1–RAGE axis playing a pivotal role in amplifying tissue damage. This study aimed to investigate the renoprotective effects of papaverine in a feces-induced peritonitis (FIP) model of sepsis and to explore its impact on HMGB1–RAGE-mediated inflammatory and oxidative pathways. *Materials and Methods*: Sepsis was induced in male Wistar rats by intraperitoneal injection of fecal slurry (1 g/kg). Animals were treated with papaverine (20 or 40 mg/kg, i.p.) one hour after FIP induction and evaluated at 24 h. Renal function (BUN, creatinine, lactate), inflammatory markers (HMGB1, TNF-α, CRP), oxidative stress (MDA), circulating sRAGE levels, renal NF-κB levels, and histopathological injury scores were assessed. *Results*: The FIP model resulted in an early mortality rate of 20% and produced marked renal histopathological alterations. Biochemically, FIP increased plasma HMGB1, TNF-α, CRP, MDA, BUN, creatinine, and lactate levels while decreasing sRAGE. Papaverine treatment dose-dependently reduced inflammatory and oxidative markers, restored sRAGE levels, improved renal function parameters, and attenuated histopathological injury. In addition, renal NF-κB levels were significantly elevated in the FIP group compared to controls and were dose-dependently reduced following papaverine treatment. *Conclusions*: FIP-induced sepsis activates an HMGB1-driven inflammatory–oxidative cascade contributing to SA-AKI. Papaverine confers dose-dependent renoprotection by suppressing HMGB1–RAGE signaling, attenuating NF-κB activation, reducing oxidative stress, and preserving renal structure and function. Targeting the HMGB1–sRAGE axis may represent a promising therapeutic strategy in sepsis-associated renal injury.

## 1. Introduction

Sepsis is a life-threatening organ dysfunction that occurs due to a dysregulated response to infection, responsible for ~49 million cases and 11 million deaths globally in 2017 [1]. Despite guideline-based bundles, substantial morbidity and mortality persist [2,3]. Sepsis-associated acute kidney injury (SA-AKI) is common and is linked to higher mortality and long-term kidney disease [4,5,6], underscoring the need for mechanism-informed adjunctive therapies.

SA-AKI may occur despite restored systemic hemodynamics, pointing to microcirculatory dysfunction and cellular stress [7,8]. Heterogeneous capillary flow and endothelial glycocalyx injury limit oxygen extraction and promote tubular hypoxia [9,10]. Inflammation is amplified by damage-associated molecular patterns; high-mobility group box 1 (HMGB1) is a late mediator released hours after stimulation and contributes to lethality [11]. HMGB1 can signal through the receptor for advanced glycation end products (RAGE) to sustain NF-κB activation, and inhibiting RAGE signaling (including via soluble decoy receptors) improves survival in polymicrobial sepsis models [12,13,14]. Clinically, circulating soluble RAGE (sRAGE) has been associated with sepsis severity and mortality [15]. Hyperlactatemia integrates hypoperfusion and stress metabolism; lactate trajectories are prognostic and lactate is recommended for guiding resuscitation [3,16,17,18].

Papaverine is an opium-derived, non-selective phosphodiesterase inhibitor that increases cAMP/cGMP and relaxes vascular smooth muscle, and may improve microvascular perfusion [19,20]. In septic shock patients on vasopressors, a single intravenous dose transiently improved sublingual microcirculation without major systemic hemodynamic changes [21]. Experimental work also suggests anti-inflammatory effects via cAMP/PKA-linked pathways and suppression of inflammatory signaling [22,23]. In feces intraperitoneal injection-based sepsis models, papaverine has been reported to modulate HMGB1/RAGE-related readouts and inflammatory markers in organ-specific settings [24,25], but its impact on polymicrobial peritonitis-driven renal injury and HMGB1/sRAGE balance remains unclear.

Feces-induced peritonitis (FIP) and related fecal slurry/fecal suspension models provide a standardized polymicrobial abdominal sepsis insult and support organ-focused phenotyping under resuscitated conditions, including early microvascular dysfunction and lactate elevations [26,27,28,29]. The present study aimed to investigate the effects of papaverine on renal function, renal histopathological injury, systemic inflammation, the HMGB1/sRAGE axis, and lactate levels in a rat FIP sepsis model.

## 2. Materials and Methods

### 2.1. Animals

A total of forty adult male Wistar albino rats (200–250 g) were included in the present experimental study. All experimental procedures were conducted in strict accordance with the principles outlined in the Guide for the Care and Use of Laboratory Animals published by the National Institutes of Health (USA). Ethical approval for the study protocol was obtained from the Institutional Animal Care and Use Committee of Science University (Approval No: 21200501).

The animals were supplied by the Experimental Animal Research Center of Science University. Throughout the experimental period, rats were maintained under standardized laboratory conditions, including a controlled ambient temperature of 22 ± 2 °C and a 12-h light/12-h dark photoperiod. Animals were housed two per cage in stainless steel cages and had unrestricted access to standard laboratory chow and tap water.

Mortality included both spontaneous death and humane endpoint-related euthanasia, defined by predefined criteria (e.g., moribund state, severe hypothermia, or ≥20% weight loss) in accordance with ARRIVE (Animal Research: Reporting of In Vivo Experiments) guidelines and AVMA (American Veterinary Medical Association) guidelines.

### 2.2. Experimental Procedures

Rats were randomly allocated into four experimental groups. Sepsis was induced in 30 animals using the feces intraperitoneal injection (FIP) model. Ten rats served as the healthy control group and received only intraperitoneal saline injection without sepsis induction.

**Figure 1:**
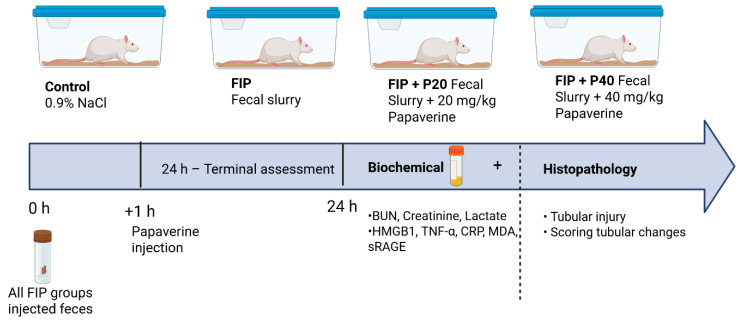
Experimental design of the feces-induced peritonitis (FIP) model and papaverine treatment protocol. Rats were allocated to control, FIP, and papaverine-treated groups (20 and 40 mg/kg). Sepsis was induced by intraperitoneal injection of fecal slurry, followed by papaverine administration at 1 h. After 24 h, biochemical and histopathological evaluations were performed to assess renal injury.

The FIP model was established as previously described by Shrum and Tyml [30,31]. FIP inoculum was prepared in an autologous manner. Immediately prior to inoculation, each rat was placed in a separate sterile collection area, and freshly excreted fecal pellets obtained by spontaneous defecation were collected from the same animal and processed individually without pooling. The pellets were transferred using sterile forceps into pre-weighed sterile tubes, and the fecal mass was recorded. Samples were diluted with sterile 0.9% NaCl to achieve the desired concentration. The fecal material was homogenized using a vortex or syringe, and after removal of large particulate matter, the suspension was administered intraperitoneally immediately after preparation without storage. The dose was calculated based on fecal mass at 1 g/kg body weight, and the injection volume was adjusted according to the final concentration of the prepared suspension. To prevent cross-contamination, each sample was prepared using separate sets of sterile consumables, and individual syringes and needles were used for each animal. A fecal slurry (1 g/kg body weight) was injected intraperitoneally (Figure 1).

Mortality within the first 24 h was recorded. Two rats died in the FIP + saline group, and one rat died in the FIP + papaverine (20 mg/kg) group. No mortality was observed in the FIP + papaverine (40 mg/kg) group. Papaverine treatment was abbreviated as PAP.

All therapeutic interventions were initiated one hour after FIP induction. The experimental period lasted 24 h.

The study groups were structured as follows:

Group 1—Control (n = 10): Non-septic rats. Received intraperitoneal 0.9% saline (1 mL/kg). No additional intervention was performed.

Group 2—FIP + Saline (n = 8): Sepsis induced by FIP. Treated with 1 mL/kg/day 0.9% NaCl intraperitoneally.

Group 3—FIP + PAP 20 mg/kg (n = 9): Sepsis induced by FIP. Treated with papaverine 20 mg/kg/day intraperitoneally (Papaverine 50 mg/2 mL, Galen).

Group 4—FIP + PAP 40 mg/kg (n = 10): Sepsis induced by FIP. Treated with papaverine 40 mg/kg/day intraperitoneally (Papaverine 50 mg/2 mL, Galen).

### 2.3. Biochemical Analysis

After 24 h, rats were anesthetized (ketamine 75 mg/kg, xylazine 15 mg/kg) and euthanized, and blood was collected by cardiac puncture. Blood samples were divided into plain tubes for serum and EDTA-containing tubes for plasma preparation. Serum samples were allowed to clot at room temperature for 30 min prior to centrifugation, whereas plasma samples collected in EDTA-containing tubes were centrifuged immediately after collection. All tubes were centrifuged at 1500× *g* for 10 min, and the supernatants (serum or plasma) were carefully separated and stored at −80 °C until biochemical analyses.

#### 2.3.1. BUN and Creatinine

Serum blood urea nitrogen (BUN) and creatinine concentrations were determined using an automated biochemical analyzer (Beckman Coulter AU2700, Beckman Coulter Inc., Brea, CA, USA) based on enzymatic colorimetric methods, according to the manufacturer’s instructions. Results were expressed in mg/dL.

#### 2.3.2. Determination of Plasma RAGE, HMGB1, TNF-α, CRP, and Lactic Acid Levels

Plasma samples prepared as described in Section 2.3 were used to determine RAGE, HMGB1, TNF-α, CRP, and lactic acid levels. Plasma TNF-α and CRP concentrations were quantified using commercially available enzyme-linked immunosorbent assay (ELISA) kits (Abcam Biosciences, Cambridge, UK). Plasma soluble receptor for advanced glycation end products (sRAGE) was measured using a rat specific ELISA kit (R&D Systems, Minneapolis, MN, USA, Cat. No: DRG00; sensitivity: 16.14 pg/mL). Plasma HMGB1 levels were determined using a commercial ELISA kit (IBL International, Hamburg, Germany, Cat. No: ST51011; sensitivity: 0.2 ng/mL). All assays were performed according to the manufacturers’ instructions. Plasma samples were diluted 1:2 prior to analysis, and TNF-α and CRP measurements were performed in duplicate; mean values were used for statistical analysis. Lactic acid levels were measured immediately after sampling using a blood gas analyzer following standard operating procedures.

#### 2.3.3. Renal Tissue Assays

Kidney samples were homogenized in ice-cold phosphate-buffered saline (pH 7.4) at a ratio of 1:5 (*w*/*v*). The homogenates were centrifuged at 5000× *g* for 15 min at 4 °C, and the supernatants were collected for subsequent analyses. Total protein concentrations were determined using the Bradford method with bovine serum albumin as the standard.

Renal tissue NF-κB levels were quantified using a commercially available rat-specific ELISA kit (MyBioSource, San Diego, CA, USA; MBS722386), according to the manufacturer’s instructions.

#### 2.3.4. Determination of Lipid Peroxidation (TBARS Assay)

Malondialdehyde (MDA) levels were determined using a thiobarbituric acid reactive substances (TBARS)-based spectrophotometric method.

To precipitate plasma proteins, each sample was mixed with two volumes of cold 10% (*w*/*v*) trichloroacetic acid and vortexed thoroughly. The mixture was centrifuged at 3000 rpm for 10 min at 4 °C, and the clear supernatant was carefully collected. An equal volume of 0.67% (*w*/*v*) thiobarbituric acid solution was added to the supernatant. The reaction mixture was incubated in a boiling water bath at 95–100 °C for 15 min under acidic conditions (pH 2–3) to allow formation of the MDA–TBA chromogen.

Following incubation, the tubes were immediately cooled on ice to terminate the reaction, then centrifuged again at 3000 rpm for 10 min to remove any residual precipitate. The absorbance of the resulting pink-colored complex was measured at 532 nm using a UV–visible spectrophotometer. MDA concentrations were calculated from a standard calibration curve generated using serial dilutions of 1,1,3,3-tetramethoxypropane and expressed as nM.

### 2.4. Histopathological Evaluation of the Kidney

#### 2.4.1. Tissue Fixation and Processing

Following deep anesthesia, blood samples were obtained via cardiac puncture. After completion of blood collection, euthanasia was ensured by exsanguination under anesthesia. Cessation of cardiac and respiratory activity was confirmed before tissue harvesting.

Immediately thereafter, kidney was carefully excised through a midline abdominal incision. The tissues were gently rinsed with cold saline to remove residual blood and promptly immersed in 4% buffered formaldehyde for fixation prior to routine histological processing.

Following anesthesia, transcardiac perfusion was performed using 200 mL of 4% buffered formaldehyde prepared in 0.1 M phosphate-buffered saline (PBS).

Kidney tissues were harvested and immersed in the same fixative solution for adequate fixation. After routine dehydration and paraffin embedding, tissue blocks were sectioned at 4 µm.

#### 2.4.2. Histological Staining

Paraffin sections were stained with hematoxylin and eosin (H&E) for general morphological evaluation. Stained slides were examined under a light microscope (Olympus BX51, Evident Corporation, Tokyo, Japan), and representative images were captured using a mounted digital camera system (Olympus C-5050, Evident Corporation, Tokyo, Japan).

#### 2.4.3. Morphometric and Semi-Quantitative Analysis

Histopathological evaluation was performed using a computerized image analysis system (Image-Pro Express version 1.4.5, Media Cybernetics, Rockville, MD, USA). For each section, ten randomly selected microscopic fields were analyzed at ×20 magnification.

All assessments were conducted by an investigator blinded to group allocation to minimize observational bias.

Renal injury was scored semi-quantitatively based on the percentage of tissue involvement, including tubular epithelial necrosis, intraluminal necrotic debris, tubular dilatation, hemorrhage, and interstitial inflammatory infiltration. The scoring system was defined as follows:

0–5% involvement: score 0;

6–20%: score 1;

21–40%: score 2;

41–60%: score 3;

61–80%: score 4;

81–100%: score 5.

This grading system was adapted from the previously described renal injury model reported by Başol et al. [32] in an experimental study of sepsis-related acute kidney injury.

### 2.5. Statistical Analyses

Statistical analyses were performed using SPSS software (version 19; IBM Corp., Armonk, NY, USA), and graphs were generated using GraphPad Prism (version 9; GraphPad Software, San Diego, CA, USA). Data are presented as mean ± SEM for biochemical variables and as median (interquartile range, IQR) for histopathological scores. Normality of data distribution was assessed using the Shapiro–Wilk test, and homogeneity of variances was evaluated with Levene’s test. For normally distributed data, one-way ANOVA was applied, followed by Tukey’s HSD post hoc test when variances were equal or Tamhane’s T2 when variances were unequal. For non-normally distributed data and ordinal variables, comparisons were performed using the Kruskal–Wallis test followed by Mann–Whitney U test for pairwise comparisons. A two-tailed *p* value of <0.05 was considered statistically significant.

#### G*Power

Sample size was determined in accordance with ARRIVE 2.0 guidelines. An a priori power analysis for a one-way ANOVA (four groups; α = 0.05; power = 0.80) was performed using G*Power software (version 3.1.9.7). The effect size was estimated based on plasma creatinine levels from a comparable rat sepsis-associated AKI study [32]. Using the reported mean ± SEM values (n = 8 per group), SEM values were converted to SD (SD = SEM × √n), and Cohen’s f was calculated, yielding a large effect size (f ≈ 1.83). This corresponded to a minimum required sample size of 9 animals per group (total N = 36). Considering the expected early mortality/attrition in polymicrobial sepsis models, particularly fecal slurry/FIP-based models [24], the group size was increased to 10 animals per group (total N = 40) to preserve statistical power at the final analysis stage while adhering to the 3Rs principle (reduction).

## 3. Results

### 3.1. Histopathological Findings

**Figure 2:**
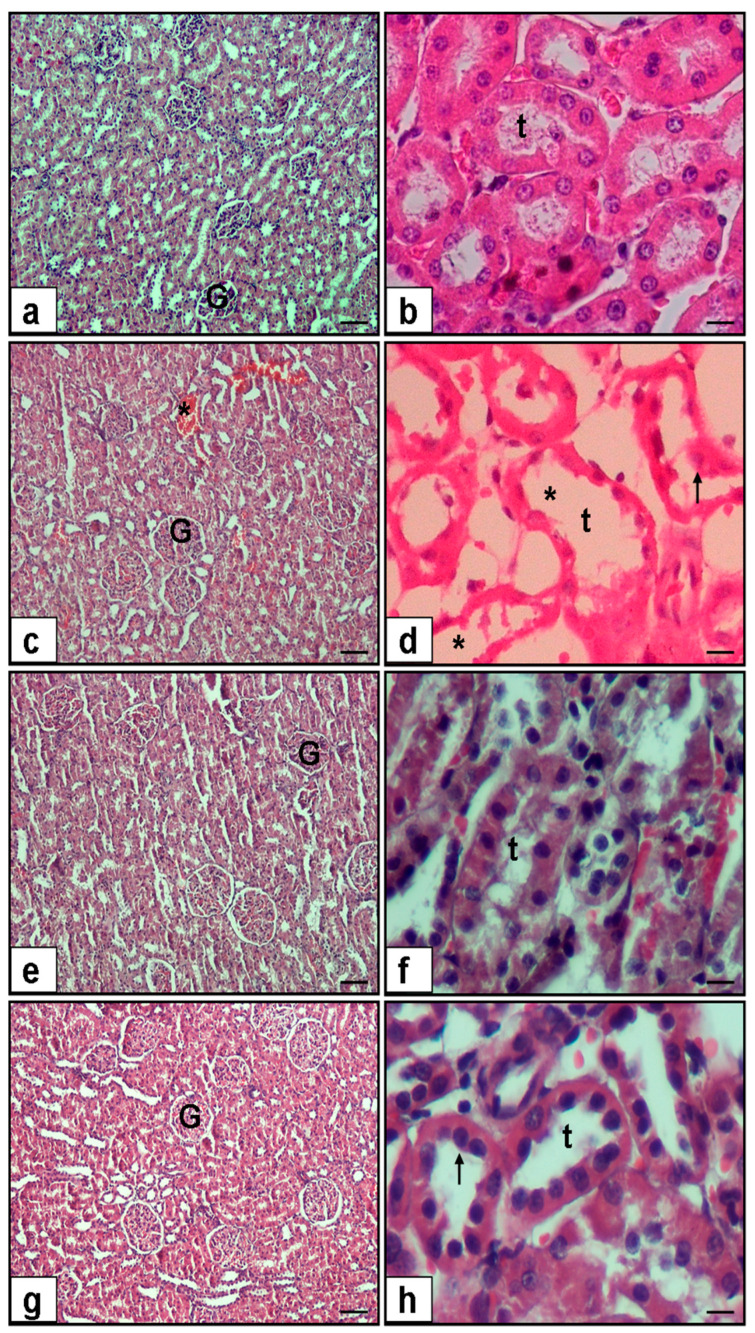
Representative hematoxylin–eosin-stained kidney sections (×10 and ×40 magnification) and semi-quantitative histopathological scoring of renal injury. (**a**,**b**) Control group showed normal renal architecture with intact glomeruli (G) and well-preserved tubular structures (t). (**c**,**d**) FIP group demonstrated marked renal injury characterized by tubular epithelial necrosis (arrow), luminal necrotic debris, tubular dilatation (*), hemorrhage, and interstitial inflammatory cell infiltration. (**e**,**f**) The FIP + PAP20 group showed partial attenuation of tubular damage, with reduced necrosis and luminal debris compared to the FIP group. (**g**,**h**) The FIP + PAP40 group exhibited substantial structural improvement, with near-normal tubular morphology and minimal interstitial inflammation. Arrow indicates tubular epithelial necrosis; asterisk (*) indicates tubular dilatation; t, renal tubules; G, glomerulus. Scale bars: ×10 and ×40 magnifications as indicated.

**Figure 3:**
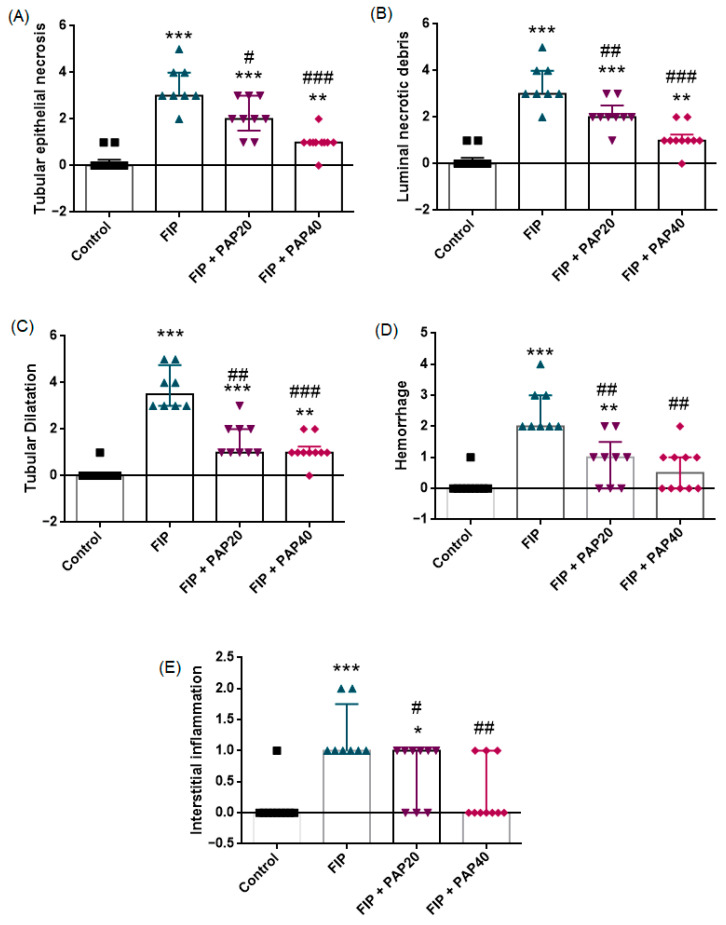
Effects of papaverine treatment on renal histopathological injury in the FIP-induced sepsis model. Semi-quantitative scores of (**A**) tubular epithelial necrosis, (**B**) luminal necrotic debris, (**C**) tubular dilatation, (**D**) hemorrhage, and (**E**) interstitial inflammation in control and experimental groups. Data are presented as median (interquartile range). Statistical analysis was performed using the Kruskal–Wallis test followed by Mann–Whitney U test for pairwise comparisons. Papaverine treatment reduced histopathological injury scores in a dose-dependent manner. * *p* < 0.05, ** *p* < 0.01, *** *p* < 0.001 vs. Control group; # *p* < 0.05, ## *p* < 0.01, ### *p* < 0.001 vs. FIP group.

The histopathological findings are presented in Figure 2 and Figure 3. Semi-quantitative histopathological analysis demonstrated significant differences among the experimental groups for tubular epithelial necrosis, luminal necrotic debris, tubular dilatation, hemorrhage, and interstitial inflammation (Kruskal–Wallis test, *p* < 0.001 for all parameters).

Compared with the control group, the FIP group exhibited significantly higher histopathological injury scores across all evaluated parameters (Mann–Whitney U test, *p* < 0.001 for tubular epithelial necrosis, luminal debris, tubular dilatation, hemorrhage, and interstitial inflammation), confirming severe renal structural damage following FIP induction.

Papaverine treatment at 20 mg/kg significantly reduced tubular epithelial necrosis (*p* = 0.015), luminal necrotic debris (*p* = 0.008), tubular dilatation (*p* = 0.001), hemorrhage (*p* = 0.002), and interstitial inflammation (*p* = 0.031) compared with the untreated FIP group.

Similarly, the 40 mg/kg dose resulted in a more pronounced reduction in tubular epithelial necrosis (*p* < 0.001), luminal necrotic debris (*p* < 0.001), tubular dilatation (*p* < 0.001), hemorrhage (*p* = 0.001), and interstitial inflammation (*p* = 0.002) relative to FIP alone.

When compared with the control group, the FIP + PAP20 group still showed significantly higher scores for tubular epithelial necrosis (*p* = 0.005), luminal necrotic debris (*p* = 0.004), tubular dilatation (*p* = 0.001), hemorrhage (*p* = 0.028), and interstitial inflammation (*p* = 0.035). Similarly, in the FIP + PAP40 group, tubular epithelial necrosis (*p* = 0.005), luminal necrotic debris (*p* = 0.004), and tubular dilatation (*p* = 0.001) remained significantly elevated compared to controls; however, hemorrhage (*p* = 0.123) and interstitial inflammation (*p* = 0.481) were not significantly different from control values. These findings suggest that the higher papaverine dose led to greater structural recovery, particularly by reducing vascular congestion and inflammatory infiltration (Figure 3).

Direct comparison between FIP + PAP20 and FIP + PAP40 groups revealed no statistically significant differences in most parameters (*p* > 0.05), suggesting that both doses provided comparable histopathological improvement, although numerically lower injury scores were observed in the higher-dose group.

### 3.2. Biochemical Finding

#### 3.2.1. Serum BUN and Creatinine

**Figure 4:**
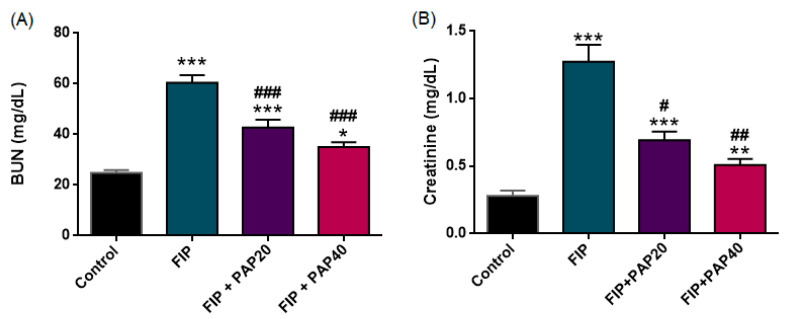
Effects of papaverine treatment on renal function parameters. (**A**) Serum blood urea nitrogen (BUN) and (**B**) serum creatinine levels in control and experimental groups. Data are expressed as mean ± SEM. Significant differences were determined by one-way ANOVA followed by Tukey’s HSD (BUN) or Tamhane’s T2 (creatinine) post hoc tests. *** *p* < 0.001 and ** *p* < 0.01 and * *p* < 0.05 versus Control; ### *p* < 0.001, ## *p* < 0.01, and # *p* < 0.05 versus FIP.

The alterations in serum BUN and creatinine levels across the experimental groups are depicted in Figure 4. Serum BUN levels differed significantly among the groups (one-way ANOVA, F (3,33) = 39.04, *p* < 0.001). BUN concentrations were markedly elevated in the FIP group compared with the control group (*p* < 0.001). Treatment with papaverine at both 20 mg/kg and 40 mg/kg significantly reduced BUN levels compared with the untreated FIP group (both *p* < 0.001). However, BUN levels in the FIP + PAP20 group remained significantly higher than those in the control group (*p* < 0.001), and similarly, BUN levels in the FIP + PAP40 group also remained elevated compared with controls (*p* = 0.016). There was no statistically significant difference between the FIP + PAP20 and FIP + PAP40 groups (*p* > 0.05).

Serum creatinine levels differed significantly among the experimental groups (one-way ANOVA, F (3,33) = 35.455, *p* < 0.001). As variance homogeneity was not met (Levene’s *p* = 0.004), Tamhane’s T2 test was applied for post hoc comparisons. Creatinine levels were significantly elevated in the FIP group compared with controls (*p* < 0.001). Papaverine treatment at both 20 mg/kg and 40 mg/kg significantly reduced creatinine levels compared with untreated FIP animals (*p* = 0.011 and *p* = 0.002, respectively). However, creatinine levels in both treatment groups remained significantly higher than those in the control group (*p* < 0.001 and *p* = 0.006, respectively), and no statistically significant difference was observed between the two treatment doses.

#### 3.2.2. Effects of Experimental Groups on Plasma MDA Levels

**Figure 5:**
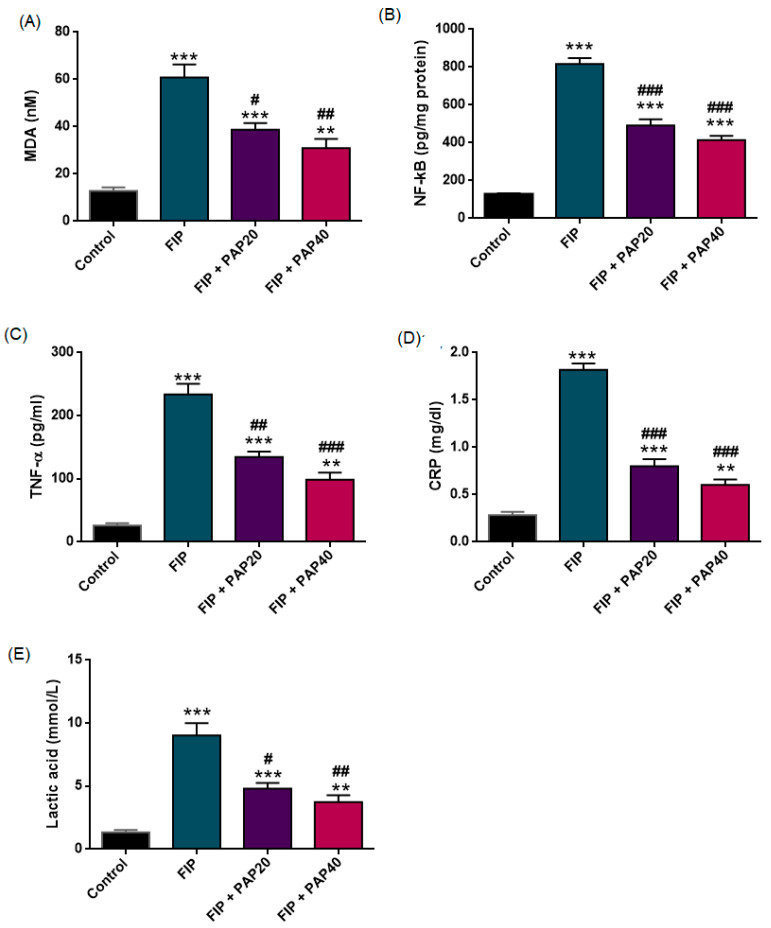
Effects of papaverine treatment on oxidative stress, inflammatory, and signaling biomarkers in the FIP-induced sepsis model. (**A**) Serum malondialdehyde (MDA), (**B**) NF-κB levels, (**C**) tumor necrosis factor-α (TNF-α), (**D**) C-reactive protein (CRP), and (**E**) lactic acid levels in control and experimental groups. Data are presented as mean ± SEM. *** *p* < 0.001, ** *p* < 0.01 versus Control; ### *p* < 0.001, ## *p* < 0.01, and # *p* < 0.05 versus FIP.

Figure 5 presents the effects of the experimental interventions on renal MDA levels. Given unequal variances (Levene’s *p* = 0.015), ANOVA with Tamhane’s T2 adjustment showed a significant group effect on MDA (F (3,33) = 29.326, *p* < 0.001).

MDA levels were significantly increased in the FIP group compared with the control group (*p* < 0.001). Treatment with papaverine at both doses significantly reduced MDA levels compared with the untreated FIP group (*p* = 0.031 and *p* = 0.004, respectively). Additionally, MDA levels in both treatment groups remained significantly higher than those in the control group (*p* < 0.001 for PAP20; *p* = 0.007 for PAP40).

#### 3.2.3. CRP and TNF-α

Plasma TNF-α, CRP and NF-κB levels of the control and experimental groups are presented in Figure 5. CRP and TNF-α levels both differed significantly among the experimental groups (CRP: F (3,33) = 119.263, *p* < 0.001; TNF-α: F (3,33) = 63.763, *p* < 0.001). Variance homogeneity was confirmed for CRP (Levene’s *p* = 0.271), and Tukey’s HSD test was applied, whereas TNF-α did not meet the homogeneity assumption (Levene’s *p* = 0.005), requiring Tamhane’s T2 post hoc analysis.

Both inflammatory markers were markedly elevated in the FIP group compared with controls (*p* < 0.001 for CRP and TNF-α). Papaverine treatment at 20 mg/kg significantly reduced CRP (*p* < 0.001) and TNF-α (*p* = 0.002) levels relative to untreated FIP animals. Similarly, the 40 mg/kg dose produced a more pronounced reduction in both CRP and TNF-α when compared with the FIP group (*p* < 0.001 for both comparisons).

Although CRP and TNF-α levels in the papaverine-treated groups remained higher than control values (CRP: PAP20 *p* < 0.001, PAP40 *p* = 0.002; TNF-α: PAP20 *p* < 0.001, PAP40 *p* = 0.001), the observed decreases—particularly at the higher dose—indicate a substantial attenuation of the systemic inflammatory response.

NF-κB levels differed significantly among the experimental groups (one-way ANOVA, F (3,33) = 130.391, *p* < 0.001). As variance homogeneity was not met (Levene’s *p* = 0.013), Tamhane’s T2 test was used for post hoc comparisons. NF-κB levels were markedly elevated in the FIP group compared with the control group (*p* < 0.001). Treatment with papaverine at both 20 mg/kg and 40 mg/kg significantly reduced NF-κB levels compared with the untreated FIP group (both *p* < 0.001). However, NF-κB levels in both treatment groups remained significantly higher than those in the control group (both *p* < 0.001), and no statistically significant difference was observed between the FIP + PAP20 and FIP + PAP40 groups (*p* = 0.352).

#### 3.2.4. Lactic Acid

A significant difference in lactic acid levels among the experimental groups was observed, as illustrated in Figure 5 (F (3,33) = 30.501, *p* < 0.001; Levene’s *p* = 0.016).

Lactate concentrations were markedly elevated in the FIP group compared with the control group (*p* < 0.001). Treatment with papaverine at 20 mg/kg significantly reduced lactate levels relative to untreated FIP animals (*p* = 0.017). A similar reduction was observed in the FIP + PAP40 group compared with FIP alone (*p* = 0.004).

Although lactate levels in both papaverine-treated groups remained significantly higher than control values (PAP20: *p* < 0.001; PAP40: *p* = 0.009), the observed decrease—particularly at the higher dose—suggests partial improvement in tissue perfusion and metabolic status.

#### 3.2.5. HMGB1 and sRAGE

**Figure 6:**
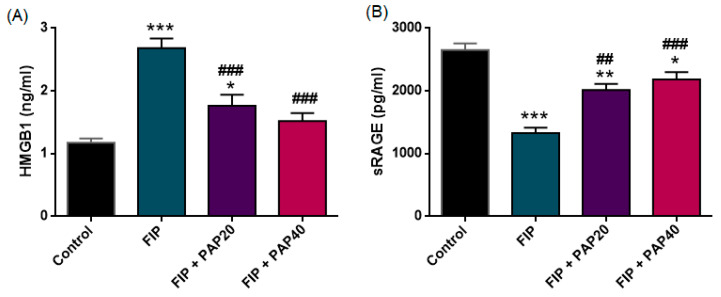
(**A**) Plasma HMGB1 levels and (**B**) plasma sRAGE levels in the experimental groups. HMGB1 levels were significantly increased in the FIP group compared with the control group (*** *p* < 0.001), whereas sRAGE levels were markedly decreased (*** *p* < 0.001). Papaverine treatment (20 and 40 mg/kg) significantly reduced HMGB1 levels compared with the FIP group (### *p* < 0.001) and partially restored sRAGE levels (## *p* < 0.01; ### *p* < 0.001). Data are presented as mean ± SEM. *** *p* < 0.001, ** *p* < 0.01, * *p* < 0.05 versus Control; ### *p* < 0.001, ## *p* < 0.01 versus FIP.

The effects of papaverine treatment on plasma HMGB1 and sRAGE levels in the Control and FIP groups are presented in Figure 6. Plasma HMGB1 and sRAGE levels both differed significantly among the experimental groups (HMGB1: F (3,33) = 21.663, *p* < 0.001; sRAGE: F (3,33) = 26.031, *p* < 0.001). Since variance homogeneity was satisfied for both parameters (HMGB1: Levene’s *p* = 0.058; sRAGE: *p* = 0.748), Tukey’s HSD test was applied for post hoc comparisons.

HMGB1 levels were significantly elevated in the FIP group compared with controls (*p* < 0.001). Both papaverine treatment groups (20 and 40 mg/kg) significantly reduced HMGB1 levels compared with untreated FIP animals (PAP20: *p* = 0.0003; PAP40: *p* < 0.0001). However, no statistically significant difference was observed between the two treatment doses (*p* = 0.5617).

Conversely, sRAGE levels were significantly lower in the FIP group than in controls (*p* < 0.001). Both papaverine-treated groups demonstrated significant increases in sRAGE levels compared with FIP alone (PAP20: *p* = 0.001; PAP40: *p* < 0.001). However, sRAGE concentrations in the treatment groups remained significantly different from control values (PAP20: *p* = 0.001; PAP40: *p* = 0.013).

## 4. Discussion

In this experimental study, we investigated the effects of papaverine on sepsis-induced renal injury in a feces-induced peritonitis model. FIP markedly increased inflammation, oxidative stress, HMGB1–RAGE axis activation, and tubular damage. Papaverine dose-dependently improved biochemical, inflammatory, and histopathological parameters, suggesting mechanistic renoprotection.

Clinical evidence indicates that mortality in sepsis and septic shock ranges from 20–30% or higher, depending on severity and comorbidities [1,33]. Experimental fecal-induced peritonitis (FIP) models show that disease severity is highly dependent on inoculum dose and observation period [26,34]. In the present study, a fecal slurry dose of 1 g/kg was administered. This dose falls within the commonly reported experimental range. The 20% early mortality observed in the FIP group indicates that the model successfully induced a clinically relevant systemic inflammatory response and early organ dysfunction consistent with sepsis.

Previous studies have shown that sepsis-associated acute kidney injury (SA-AKI) is primarily characterized by acute tubular damage [35,36]. Experimental CLP and fecal peritonitis models have shown that tubular epithelial necrosis, luminal cast formation, microvascular congestion, and inflammatory infiltration are dominant findings [4,37,38]. Consistent with these observations, the present study found that the FIP group exhibited marked tubular epithelial necrosis, luminal necrotic debris, tubular dilatation, hemorrhage, and interstitial inflammation. These structural alterations were accompanied by significant elevations in serum BUN and creatinine, indicating impaired glomerular filtration. Increased lactate levels were also observed. Numerous studies have shown that hyperlactatemia reflects microcirculatory dysfunction and tissue dysoxia in sepsis [16,39]. Persistent lactate elevation has been associated with worse renal outcomes and increased mortality [40,41]. Thus, our biochemical and histopathological findings are consistent with the established SA-AKI framework.

Previous experimental studies have consistently demonstrated that inflammation and oxidative stress represent central drivers of tissue injury in acute kidney injury (AKI), particularly in sepsis-associated AKI [42,43,44,45]. HMGB1 is a nuclear protein involved in chromatin regulation, but under stress or sepsis, it is released extracellularly, where it acts as a damage-associated molecular pattern (DAMP) [46,47]. Extracellular HMGB1 primarily signals through TLR4 and RAGE receptors. Activation of this axis amplifies NF-κB–mediated pro-inflammatory cytokine responses. In renal experimental models, neutralization of HMGB1 has been shown to attenuate tubular injury and reduce inflammatory mediators such as IL-6 and TNF-α [46,48,49]. Similarly, increased HMGB1 levels have been reported in feces-induced peritonitis and CLP models in vivo [24,50,51], supporting HMGB1-mediated inflammation in sepsis. In the present study, plasma HMGB1, TNF-α, and CRP levels were significantly increased in the FIP group, accompanied by a significant increase in renal NF-κB levels, consistent with previous experimental studies demonstrating activation of HMGB1-associated inflammatory signaling in sepsis.

HMGB1 also exacerbates renal injury through oxidative mechanisms. Previous studies indicated that HMGB1 increases mitochondrial ROS production and promotes tubular epithelial cell death [52,53]. HMGB1–TLR4 interaction in endothelial and immune cells further amplifies ROS generation, a positive feedback loop [54]. In addition, HMGB1 translocation has been associated with increased lipid peroxidation, reflected by elevated MDA levels [53]. Lipid peroxidation disrupts membrane integrity and contributes to tubular epithelial injury and renal dysfunction [44,45]. Numerous polymicrobial sepsis models have demonstrated increased MDA, accompanied by depletion of antioxidant defenses such as GSH, SOD, and CAT [42,43,44]. Similarly, in the present study, FIP-induced sepsis significantly increased plasma MDA levels. Serum BUN and creatinine were also elevated. Our data suggest that oxidative stress is mechanistically linked to structural kidney damage in FIP sepsis.

In the present study, sRAGE levels significantly decreased in the FIP group. sRAGE acts as a soluble “decoy” receptor that binds HMGB1 and limits activation of membrane-bound RAGE signaling [49,55,56]. Thus, decreased sRAGE levels may reduce ligand-scavenging capacity, allowing greater HMGB1–RAGE interaction and thereby amplifying downstream inflammatory signaling, including increased production of pro-inflammatory mediators such as TNF-α and CRP. Consistent with this mechanism, experimental sepsis-AKI models have shown that exogenous sRAGE administration attenuates elevations in BUN and creatinine and suppresses RAGE-related MyD88/MAPK–NF-κB signaling, thereby mitigating renal injury [57]. The pattern of HMGB1 elevation together with sRAGE reduction may reflect a shift toward enhanced pro-inflammatory RAGE signaling.

Papaverine treatment dose-dependently reduced levels of HMGB1, TNF-α, CRP, MDA, BUN, creatinine, and lactate. Histopathological injury scores also improved significantly. Previous experimental studies suggest that papaverine may modulate HMGB1–RAGE signaling and reduce oxidative stress markers [24,58]. As a non-selective phosphodiesterase inhibitor, papaverine increases intracellular cAMP and induces vasodilation. Improved microvascular perfusion may reduce tissue hypoxia and downstream ROS production (Damiani et al., 2023 [59]). The restoration of sRAGE levels observed in our study further supports modulation of the HMGB1–RAGE axis.

Papaverine was administered 1 h after FIP induction to model an early post-insult therapeutic intervention rather than prophylaxis. Polymicrobial peritonitis rapidly initiates systemic inflammatory signaling following fecal inoculation [60]. Current sepsis guidelines also recommend initiating treatment as early as possible [3]. The 24-h timepoint was selected to capture the early phase of sepsis-associated organ injury, when inflammatory activation and microcirculatory dysfunction are most pronounced. The FIP model consistently induces systemic inflammation and organ injury within this period. Clinical manifestations may evolve over 24–72 h [61], and early modulation of inflammatory pathways may therefore influence subsequent injury outcomes. Delayed-dosing studies are needed to better define the therapeutic window of papaverine.

Our findings have potential translational relevance. Sepsis-associated AKI frequently occurs despite restoration of systemic hemodynamics, highlighting the importance of microcirculatory dysfunction and tissue hypoxia [7]. In this context, papaverine’s vasodilatory and phosphodiesterase-inhibiting properties may improve microvascular perfusion and attenuate tissue hypoxia. Supporting this concept, a clinical study demonstrated that papaverine transiently improves sublingual microcirculation in patients with septic shock without major systemic hemodynamic effects [21]. Furthermore, modulation of the HMGB1–RAGE axis observed in our study aligns with growing evidence identifying this pathway as a promising therapeutic target in sepsis-related organ injury. Collectively, these findings suggest that papaverine may represent a mechanistically plausible adjunctive therapy targeting microcirculatory and inflammatory dysregulation in sepsis-associated AKI. Therefore, well-designed clinical studies are required to determine its safety, optimal dosing, therapeutic window, and efficacy in human sepsis. However, although the FIP model captures key features of clinical sepsis, its ability to fully replicate the complexity of human disease remains limited.

### Limitations

This study has several limitations that should be acknowledged. The absence of a papaverine-only control group without FIP induction limits the ability to distinguish between the direct pharmacological effects of papaverine and its protective effects under septic conditions. Standard supportive interventions such as fluid resuscitation, antibiotic administration, and hemodynamic monitoring were not implemented in this experimental model in order to isolate the direct biological effects of papaverine; while this design may limit direct clinical translation, it allows clearer interpretation of the intrinsic pharmacological and mechanistic effects of papaverine under septic conditions. Only male rats were included to minimize variability associated with hormonal fluctuations; therefore, potential sex-related differences in inflammatory and oxidative stress responses could not be evaluated. The relatively short observation period (24 h) also limits the assessment of long-term outcomes and recovery processes in sepsis-associated acute kidney injury, and future studies including longer follow-up periods and both sexes are warranted. Evaluation of additional pathways such as MAPK or related molecular mediators would further strengthen the mechanistic framework; however, this is acknowledged as a limitation of the present study. Future investigations of these pathways may provide deeper insight into the molecular mechanisms underlying papaverine’s effects.

## 5. Conclusions

The present study demonstrates that feces-induced peritonitis (FIP)-mediated sepsis triggers a coordinated inflammatory and oxidative cascade characterized by increased HMGB1 release, elevated TNF-α and CRP levels, increased renal NF-κB activation, enhanced lipid peroxidation (MDA), and a concomitant reduction in circulating sRAGE. This imbalance was accompanied by significant renal functional impairment, as reflected by increased BUN and creatinine levels, and by marked histopathological alterations, including tubular epithelial necrosis, luminal debris accumulation, tubular dilatation, hemorrhage, and interstitial inflammation.

Importantly, papaverine administration attenuated both biochemical and structural markers of renal injury in a dose-dependent manner. Treatment was associated with suppression of HMGB1; NF-κB and TNF-α elevations; reduced oxidative stress; partial restoration of sRAGE levels; and significant improvement in tubular architecture. These findings suggest that modulation of the HMGB1–RAGE axis and downstream inflammatory signaling may play a central role in limiting sepsis-associated acute kidney injury (SA-AKI).

Collectively, our results support the concept that restoring the balance between HMGB1/NF-κB/TNF-α activation and sRAGE-mediated ligand neutralization, together with improving microcirculatory and inflammatory dysregulation, represents a biologically plausible therapeutic strategy to reduce renal vulnerability in polymicrobial sepsis.

## Data Availability

The data that support the findings of this study are not publicly available due to ethical reasons, but are available from the corresponding author upon request.
